# Job vacancy at the European Centre for Disease Prevention and Control

**DOI:** 10.2807/1560-7917.ES.2017.22.21.30538

**Published:** 2017-05-25

**Authors:** Eurosurveillance editorial team

**Affiliations:** 1 European Centre for Disease Prevention and Control (ECDC), Stockholm, Sweden

The European Centre for Disease Prevention and Control (ECDC) invites applications for the following position:

**Deputy Head of Unit, Surveillance and Response Support Unit**

The deadline for the application is 20 June 2017. For more information, visit the ECDC website:

http://ecdc.europa.eu/en/aboutus/jobs/Pages/JobOpportunities.aspx

